# Comparison of nasojejunal nutrition and intravenous nutrition supplementation in patients with upper gastrointestinal tract strictures and analysis of risk factors for malnutrition

**DOI:** 10.1097/MD.0000000000038820

**Published:** 2024-07-05

**Authors:** Yunfei Yang, Huirong Ji, Guangyong Shi

**Affiliations:** a Department of radiology, Peking University First Hospital—Miyun Hospital, Beijing, China.

**Keywords:** intravenous nutrition, malnutrition, nasojejunal nutrition, risk factors

## Abstract

This study examines the effectiveness of nasojejunal and intravenous nutrition in supplementing nutrition for patients with upper gastrointestinal (GI) strictures and analyzes the risk factors associated with malnutrition to provide references for clinical nutrition strategies. A retrospective analysis was conducted on 71 patients with upper GI strictures caused by esophageal and gastric cancers, who received nutritional support from January 2015 to January 2023. Out of these, 53 patients had complete baseline and follow-up data. We collected general clinical and perioperative data for comparison of the efficacy between nasojejunal nutrition and intravenous nutrition. Risk factors for malnutrition were analyzed using univariate and multivariate logistic regression. Malnutrition occurred in 24.53% (13/53) of the patients with upper GI strictures. The incidence of malnutrition was 6.06% (2/33) in the nasojejunal nutrition group compared to 55.00% (11/20) in the intravenous nutrition group, with a statistically significant difference (*P* < .001). Univariate and multivariate regression analyses identified diabetes (*P* < .001), initial blood K (*P* = .011), pathological staging (*P* < .001), and pathological grading (*P* < .001) as risk factors for malnutrition in patients with upper GI strictures. Diabetes (*P* = .028), initial blood K (*P* = .018), and pathological staging (*P* = .011) were found to be independent risk factors. Nasojejunal nutrition results in a lower incidence of malnutrition compared to intravenous nutrition in patients with upper GI strictures. Diabetes, initial blood K, pathological staging, and pathological grading are risk factors for malnutrition, with diabetes, initial blood K, and pathological staging serving as independent risk factors.

## 1. Introduction

Esophageal and gastric cancers rank seventh and fifth respectively among all cancers globally, with even higher incidence rates in East Asian countries.^[1–3]^ Due to indistinct early symptoms, these cancers are often diagnosed at advanced stages with complications. Upper gastrointestinal (GI) strictures, as a common complication, adversely affect the patients’ ability to eat and receive nutritional supplementation.

Nutritional support for patients with upper GI strictures caused by gastric and esophageal cancers is a challenge, as poor nutrition cannot sustain subsequent systemic chemotherapy.^[4]^ Malnutrition is a risk factor for poor adherence to chemotherapy and radiotherapy, ultimately affecting tumor prognosis.^[5,6]^ Nutritional delivery methods are mainly divided into enteral and parenteral nutrition. In cases of upper GI strictures, parenteral nutrition is primarily provided intravenously, while enteral nutrition is mainly administered through nasogastric and nasojejunal tubes.^[7,8]^

Enteral and parenteral nutrition each have their appropriate scenarios and specific advantages and disadvantages.^[9,10]^ Generally, enteral nutrition is more akin to natural eating because it delivers nutrients through the digestive tract.^[11]^ Conversely, parenteral nutrition directly transports nutrients into the bloodstream.^[12]^ This study aims to explore the differences in the effectiveness of nasojejunal and intravenous nutrition in supplementing nutrition for patients with upper GI strictures, providing a reference for clinicians in selecting nutritional supplementation methods. Additionally, this study analyzes risk factors for malnutrition in patients with upper GI strictures to guide clinical nutritional strategies.

## 2. Methods

A total of 71 patients with upper GI strictures caused by esophageal and gastric cancers received nutritional support treatment at the First Hospital of Peking University-Miyun Hospital, between January 2015 and January 2023. Among these, 53 patients had complete baseline and follow-up data.

Within this group, 20 patients received intravenous nutritional support, and 33 underwent nasojejunal nutrition. Clinical data and follow-up information were collected, including gender, age, body mass index (BMI), smoking status, presence of hypertension, diabetes, coronary heart disease, pathological staging, pathological grading, initial albumin, initial blood Na, initial blood K, initial hemoglobin, albumin after 1 week, blood Na after 1 week, blood K after 1 week, hemoglobin after 1 week, albumin after 1 month, blood Na after 1 month, blood K after 1 month, and hemoglobin after 1 month. The period of 1 week and 1 month was calculated from the start of intravenous supplementation or nasojejunal nutrition. Follow-up data included the percentage of weight loss 1 week and 1 month later. A weight loss of over 1% within a week or over 5% within a month from the start of nutritional supplementation was defined as malnutrition.^[13]^ The percentage of weight loss was calculated as (current weight − initial weight)/initial weight × 100%.

Inclusion criteria: Patients with upper GI strictures caused by esophageal and gastric cancer; patients with complete clinical and follow-up data.

### 2.1. Exclusion criteria: Patients who can eat normally; patients with widespread metastatic tumors

This study was conducted in accordance with the principles of the Declaration of Helsinki (2013 revised version), and individual consent for this retrospective analysis was waived.

### 2.2. Nasojejunal nutrition procedure

Understand the patient medical history, assess the nasal structure and GI tract condition. Place the patient in a semi-recumbent position, keeping the head and neck slightly forward. Select the appropriate diameter and length of the feeding tube, apply lubricant to the tube, and slowly insert it into 1 nostril. Gradually guide the tube through the nasopharynx, esophagus, and into the stomach or duodenum. Typically, insert into the stomach first, then guide into the jejunum. During this process, the patient may swallow water or perform slight coughing to assist in guiding. Use X-ray, air insufflation auscultation, or inject a small amount of liquid to confirm the tube position by observing if there is gastric content reflux. Fix the tube at the nostril or cheek to prevent displacement. Inject the nutrient solution into the infusion set, and input the nutrient solution at a set flow rate or by pump.

### 2.3. Follow up

Some patients were regularly visited at the clinic to collect data on weight, albumin, hemoglobin, blood Na, and blood K, while others had their data collected via telephone follow-up.

### 2.4. Statistical analysis

Statistical analysis was performed using SPSS 22.0 (IBM Corp., Armonk, NY, USA). Quantitative variables included age, BMI, initial albumin, initial blood Na, initial blood K, initial hemoglobin, albumin after 1 week, blood Na after 1 week, blood K after 1 week, hemoglobin after 1 week, albumin after 1 month, blood Na after 1 month, blood K after 1 month, and hemoglobin after 1 month. Qualitative variables included gender, smoking status, presence of hypertension, diabetes, coronary heart disease, pathological staging, and pathological grading. Data that followed a normal distribution were expressed as mean ± standard deviation, while skewed data were described using the median. For continuous variables, those following a normal distribution were analyzed using the t-test, and those not following a normal distribution were analyzed using the Mann–Whitney *U* test. Categorical variables were analyzed using Fisher exact probability test. Univariate and multivariate regression analyses were used to determine the independent risk factors for malnutrition in patients with upper GI strictures. A *P* value < .05 was considered statistically significant.

## 3. Results

### 3.1. Baseline patient data

Baseline data of the patients are shown in Table 1. A total of 53 patients with upper GI strictures were included in this study, with 62.26% (33/53) receiving nasojejunal nutrition and 37.74% (20/53) receiving intravenous nutrition. Among these, malnutrition occurred in 24.53% (13/53) of the patients after nutritional supplementation.

**Table 1:** Basic characteristics of the patients.

Variable	Mean (SD) or n/N
Patients	53
Mean age (y)	52.02 ± 14.16
BMI (kg/m^2^)	24.99 ± 0.07
Sex, n (%)	
Male	41 (77.36)
Female	12 (22.64)
Hypertension, n (%)	
Yes	10 (18.87)
No	43 (81.13)
Diabetes mellitus, n (%)	
Yes	12 (22.64)
No	41 (77.36)
CHD, n (%)	
Yes	7 (13.21)
No	46 (86.79)
Smoking, n (%)	
Yes	17 (32.08)
No	36 (67.92)
Pathological T, n (%)	
T1–T2	30 (56.60)
T3–T4	23 (43.40)
Histological grade, n (%)	
G1–G2	28 (52.83)
G3–G4	25 (47.17)
Initial albumin (g/L)	36.21 ± 7.58
Initial blood Na (mmol/L)	138.01 ± 17.30
Initial blood K (mmol/L)	3.98 ± 0.29
Initial hemoglobin (g/L)	140.60 ± 14.36
Albumin after a wk (g/L)	32.17 ± 6.83
Blood Na after a wk (mmol/L)	137.96 ± 17.57
Blood K after a wk (mmol/L)	3.90 ± 0.35
Hemoglobin after a wk (g/L)	124.21 ± 14.47
Albumin after a mo (g/L)	29.15 ± 6.76
Blood Na after a mo (mmol/L)	132.73 ± 17.61
Blood K after a mo (mmol/L)	3.51 ± 0.43
Hemoglobin after a mo (g/L)	114.21 ± 15.41
Nutritional supplementation methods, n (%)	
Nasojejunal nutrition	33 (62.26)
Intravenous nutrition	20 (37.74)
Malnutrition, n (%)	
Yes	13 (24.53)
No	40 (75.47)

BMI = body mass index; CHD = coronary heart disease.

### 3.2. Analysis of risk factors for malnutrition in patients with GI strictures

Clinical data for the normal nutrition group and the malnutrition group are presented in Table 2. The rate of diabetes in the normal nutrition group was 12.50% (5/40), compared to 53.85% (7/13) in the malnutrition group, showing a statistically significant difference (*P* = .002).

**Table 2:** Comparing the difference between normal nutrition group and malnutrition group.

Variable	Normal nutrition group	Malnutrition group	*P* value
Patients	40	13	
Mean age (y)	50.78 ± 14.16	55.13 ± 13.70	.657
BMI (kg/m^2^)	24.52 ± 4.25	26.19 ± 2.75	.206
Sex, n (%)			.619
Male	31 (77.50)	10 (76.92)	
Female	9 (22.50)	3 (23.08)	
Hypertension, n (%)			.655
Yes	7 (17.50)	3 (23.08)	
No	33 (82.50)	10 (76.92)	
Diabetes mellitus, n (%)			.002
Yes	5 (12.50)	7 (53.85)	
No	35 (87.50)	6 (46.15)	
CHD, n (%)			.499
Yes	6 (15.00)	1 (7.69)	
No	34 (85.00)	12 (92.31)	
Smoking, n (%)			.908
Yes	13 (32.50)	4 (30.77)	
No	27 (67.50)	9 (69.23)	
Pathological T, n (%)			<.001
T1–T2	29 (72.50)	1 (7.69)	
T3–T4	11 (27.50)	12 (92.31)	
Histological grade, n (%)			.002
G1–G2	26 (65.00)	2 (15.38)	
G3–G4	14 (35.00)	11 (84.62)	
Initial albumin (g/L)	36.68 ± 6.90	35 ± 8.96	.923
Initial blood Na (mmol/L)	137.29 ± 20.34	139.82 ± 2.13	.671
Initial blood K (mmol/L)	3.91 ± 0.26	4.15 ± 0.30	.01
Initial hemoglobin (g/L)	143.29 ± 14.67	133.29 ± 20.43	.094
Albumin after a wk (g/L)	32.565.99	31.28.54	.992
Blood Na after a wk (mmol/L)	137.16 ± 20.76	139.93 ± 1.21	.679
Blood K after a wk (mmol/L)	3.84 ± 0.38	1.05 ± 0.23	.059
Hemoglobin after a wk (g/L)	125 ± 13.90	122.07 ± 15.73	.354
Albumin after a mo (g/L)	30.29 ± 5.68	26.27 ± 8.24	.964
Blood Na after a mo (mmol/L)	133.16 ± 20.78	131.66 ± 2.96	.410
Blood K after a mo (mmol/L)	3.62 ± 0.39	3.27 ± 0.41	.014
Hemoglobin after a mo (g/L)	115.05 ± 15.53	111.93 ± 14.86	.351
Nutritional supplementation methods, n (%)			<.001
Nasojejunal nutrition	31 (77.50)	2 (15.38)	
Intravenous nutrition	9 (22.50)	11 (84.62)	

BMI = body mass index; CHD = coronary heart disease.

The rate of lower pathological staging in the normal nutrition group was 72.50% (29/40), compared to 7.69% (1/13) in the malnutrition group, with a statistically significant difference (*P* < .001). The rate of lower pathological grading in the normal nutrition group was 65.00% (26/40), compared to 15.38% (2/13) in the malnutrition group, with a statistically significant difference (*P* = .002). The initial blood K level in the normal nutrition group was 3.91 ± 0.26 mmol/L, compared to 4.15 ± 0.30 mmol/L in the malnutrition group, showing a statistically significant difference (*P* = .01). One month later, the blood K level in the normal nutrition group was 3.62 ± 0.39 mmol/L, compared to 3.27 ± 0.41 mmol/L in the malnutrition group, showing a statistically significant difference (*P* = .014). The rate of nasojejunal nutrition in the normal nutrition group was 77.50% (31/40), compared to 15.38% (2/13) in the malnutrition group, with a statistically significant difference (*P* < .001).

Univariate and multivariate regression analyses found that diabetes (*P* < .001), initial blood K (*P* = .011), pathological staging (*P* < .001), and pathological grading (*P* < .001) were risk factors for malnutrition in patients with upper GI strictures. Diabetes (*P* = .028), initial blood K (*P* = .018), and pathological staging (*P* = .011) were independent risk factors for malnutrition (Table 3).

**Table 3:** Univariate analysis and multivariate analysis of malnutrition in patients with upper gastrointestinal tract strictures.

	Univariate analysis		Multivariate analysis	
Characteristic	HR (95% CI)	*P* value	HR (95% CI)	*P* value
Smoking, yes vs no	0.359 (0.380–5.386)	.597		
Hypertension, yes vs no	0.662 (0.460–8.175)	.367		
Diabetes, yes vs no	1.754 (0.043–0.691)	<.001	0.049 (0.003–0.722)	.028
CHD, yes vs no	0.965 (0.042–3.466)	.392		
Initial albumin	0.03 (0.894–1.0553)	.468		
Initial blood Na	0.013 (0.955–1.074)	.664		
Initial blood K	3.266 (2.1–327)	.011	15.789 (2.293–81.989)	.018
Initial hemoglobin	0.051 (0.905–1.998)	.137		
BMI	0.106 (0.951–1.299)	.183		
Age	0.022 (0.979–1.069)	.249		
Sex, male vs female	0.795 (0.117–1.744)	.619		
Pathological T, T3 + T4 vs T1 + T2	3.809 (5.191–392.029)	<.001	0.011 (0.000–0.350)	.011
Histological grade, G1 + G2 vs G3 + G4	2.645 (2.736–72.480)	<.001	0.559 (0.051–6.146)	.634

BMI = body mass index; CHD = coronary heart disease.

### 3.3. Comparison of the efficacy of nasojejunal and intravenous nutrition supplementation in patients with upper GI strictures

Clinical data for the nasojejunal nutrition group and the intravenous nutrition group are shown in Table 4. The rate of malnutrition caused by nasojejunal nutrition in patients with upper GI strictures was 6.06% (2/33), compared to 55.00% (11/20) in the intravenous nutrition group, showing a statistically significant difference (*P* < .001). One month later, the blood K level in the nasojejunal nutrition group was 3.67 ± 0.39 mmol/L, compared to 3.28 ± 0.36 mmol/L in the intravenous nutrition group, showing a statistically significant difference (*P* = .004). The rate of lower clinical staging in the nasojejunal nutrition group was 69.70% (23/33), compared to 35.00% (7/20) in the intravenous nutrition group, with a statistically significant difference (*P* = .013).

**Table 4:** Comparison of efficacy of nasojejunal nutrition and intravenous nutrition in patients with upper gastrointestinal tract strictures.

Variable	Nasojejunal nutrition group	Intravenous nutrition group	*P* value
Patients	33	20	
Mean age (y)	51.33 ± 14.37	53.15 ± 13.75	.651
BMI (kg/m^2^)	24.73 ± 4.41	25.42 ± 3.02	.539
Sex, n (%)			.177
Male	26 (78.79)	15 (75.00)	
Female	7 (21.21)	5 (25.00)	
Hypertension, n (%)			.374
Yes	5 (15.15)	5 (25.00)	
No	28 (84.85)	15 (75.00)	
Diabetes mellitus, n (%)			.094
Yes	5 (15.15)	7 (35.00)	
No	28 (84.85)	13 (65.00)	
CHD, n (%)			.169
Yes	6 (18.18)	1 (5.00)	
No	27 (81.82)	19 (95.00)	
Smoking, n (%)			.801
Yes	11 (33.33)	6 (30.00)	
No	22 (66.67)	14 (70.00)	
Pathological T, n (%)			.013
T1–T2	23 (69.70)	7 (35.00)	
T3–T4	10 (30.30)	13 (65.00)	
Histological grade, n (%)			.374
G1–G2	19 (57.58)	9 (45.00)	
G3–G4	14 (42.42)	11 (55.00)	
Initial albumin (g/L)	35.64 ± 5.32	37.15 ± 10.20	.483
Initial blood Na (mmol/L)	136.88 ± 21.80	139.86 ± 1.93	.592
Initial blood K (mmol/L)	3.95 ± 0.27	4.023 ± 0.32	.394
Initial hemoglobin (g/L)	142.39 ± 14.47	137.47 ± 13.61	.242
Albumin after a wk (g/L)	31.455 ± 4.73	33.35 ± 9.20	.333
Blood Na after a wk (mmol/L)	136.64 ± 22.28	140.07 ± 1.26	.575
Blood K after a wk (mmol/L)	3.90 ± 0.38	3.908 ± 0.31	.949
Hemoglobin after a wk (g/L)	126.24 ± 13.74	120.68 ± 14.69	.188
Albumin after a mo (g/L)	29.09 ± 4.45	29.25 ± 9.39	.934
Blood Na after a mo (mmol/L)	132.77 ± 22.31	132.67 ± 3.15	.957
Blood K after a mo (mmol/L)	3.67 ± 0.39	3.28 ± 0.36	.004
Hemoglobin after a mo (g/L)	116.88 ± 15.19	1099.58 ± 14.69	.107
Malnutrition, n (%)			<.001
Yes	2 (6.06)	11 (55.00)	
No	31 (93.94)	9 (45.00)	

BMI = body mass index; CHD = coronary heart disease.

## 4. Discussion

Nutritional status is one of the key factors for subsequent treatment in patients with gastric and esophageal cancers. This study compared the effectiveness of nutritional supplementation methods for patients with upper GI strictures who are unable to eat normally. It was found that nasojejunal nutrition results in a lower incidence of poor prognosis compared to intravenous nutrition. The analysis of risk factors for malnutrition identified diabetes and weight as independent risk factors for malnutrition in patients with upper GI strictures.

While few studies have shown that malnutrition affects subsequent chemotherapy in gastric and esophageal cancers, numerous studies indicate that patients with lower nutritional indices who undergo surgery or chemotherapy have higher rates of complications and mortality.^[14–16]^ Good nutritional status is key to maintaining normal immune function. Malnutrition leads to a decline in immune system function, making patients more susceptible to infections, which may be a reason why poor nutrition is associated with higher rates of complications and mortality in surgery or chemotherapy. Additionally, malnourished patients may not tolerate normal doses of chemotherapy drugs, necessitating dose adjustments or extended treatment periods, which can reduce the effectiveness of treatment. Appropriate nutritional supplementation is particularly important for subsequent treatment, and clinicians’ understanding of different nutritional supplementation methods helps them adjust patients’ nutritional status and thus improve treatment outcomes.

This study found diabetes (*P* < .001), initial blood K levels (*P* = .011), pathological staging (*P* < .001), and pathological grading (*P* < .001) as risk factors for malnutrition in patients with upper GI strictures, with diabetes (*P* = .028), initial blood K (*P* = .018), and pathological staging (*P* = .011) as independent risk factors. Rajamanickam et al ^[17]^ found that diabetes is related to malnutrition and may be associated with significant regulation of blood glucose, hormones, and cytokines. Vural Keskinler et al ^[18]^ in a cross-sectional study found that among obese and diabetic patients, 1 in 7 is at risk of malnutrition. Ding et al ^[19]^ identified age (*P* = .013), BMI (*P* = .001), anemia (*P* = .005), pre-albumin (*P* = .010), albumin (*P* = .002), tumor location (*P* = .001), and tumor size (*P* = .002) as risk factors for malnutrition in patients with GI stromal tumors. Diabetic patients often have insulin resistance and persistent hyperglycemia, which interferes with the body normal metabolism of proteins, fats, and carbohydrates, possibly leading to malnutrition. Low potassium can affect muscle and nerve function, causing fatigue, muscle weakness, and even heart problems, thereby affecting the overall nutritional status and health of the individual. High-stage tumors may lead to higher energy and nutritional needs, and the tumor itself may consume a large amount of nutrients, causing the so-called tumor cachexia, which may be a cause of malnutrition.^[20–22]^ High-grade tumors usually grow faster and have increased demands for energy and nutrients. The high metabolic activity of tumor cells consumes large amounts of glucose and other nutrients, thereby reducing the nutrient supply to normal cells.^[23,24]^ For patients with high-stage, high-grade tumors, clinicians should pay more attention to their nutritional status and appropriately increase nutritional supplementation.

This study found that the incidence of malnutrition after nasojejunal nutrition (2/33, 6.06%) was lower than that after intravenous nutrition (11/20, 55.00%), with a statistically significant difference (*P* < .001). Enteral nutrition is more consistent with the body natural digestion and absorption process. This method helps maintain the function and integrity of the intestines. At the same time, enteral nutrition also has certain advantages in terms of cost. Nasojejunal nutrition may be a better clinical choice for patients with upper GI strictures caused by gastric and esophageal cancers.

This study has certain limitations. First, the sample size of this study is relatively small, which may introduce some bias in the analysis. Additionally, as the study was conducted at a single center and lacks multi-center data, this may limit the general applicability of the study results.

## Author contributions

**Conceptualization:** Yunfei Yang, Huirong Ji, Guangyong Shi.

**Data curation:** Yunfei Yang, Huirong Ji, Guangyong Shi.

**Formal analysis:** Yunfei Yang, Huirong Ji, Guangyong Shi.

**Investigation:** Guangyong Shi.

**Methodology:** Guangyong Shi.

**Project administration:** Yunfei Yang.

**Resources:** Yunfei Yang, Guangyong Shi.

**Software:** Yunfei Yang, Guangyong Shi.

**Supervision:** Yunfei Yang.

**Writing – original draft:** Yunfei Yang, Huirong Ji.

**Writing – review & editing:** Yunfei Yang, Guangyong Shi.
